# Effect of best bet methane abatement feed on feed intake, digestibility, live weight change, and methane emission in local Menz breed sheep in Ethiopia

**DOI:** 10.3389/fvets.2025.1538758

**Published:** 2025-02-14

**Authors:** Wondimagegne Bekele, Abiy Zegeye, Addis Simachew, Nobuyuki Kobayashi

**Affiliations:** ^1^Department of Applied Animal Science and Welfare, Swedish University of Agricultural Sciences, Umeå, Sweden; ^2^Institute of Biotechnology, Addis Ababa University, Addis Ababa, Ethiopia; ^3^Arid Land Research Center, Tottori University, Tottori, Japan; ^4^Shikoku Center, Japan International Cooperation Agency, Tokyo, Japan

**Keywords:** condensed tannins, laser methane detector, local sheep, methane emission, modeling, protein efficiency ratio, *Ziziphus spina-christi*

## Abstract

This study continued the *in vitro* screening of locally available ruminant feedstuffs for optimum nutrient composition and low methane (CH_4_) production in Ethiopia. The best bet feeds from the *in vitro* study, hereafter called the test feeds, include dried leaves of *Acacia nilotica*, *Ziziphus spina-christi*, and brewery spent grains (BSG). The study involves four treatments: Control, Acacia, BSG, and Ziziphus; each treatment provided an equivalent crude protein and estimated enteric CH_4_ emissions using Modeling and a Laser CH_4_ detector (LMD). The experiment was designed as a randomized complete block, using initial weight as the blocking factor for 21 yearling castrated Menz sheep. The study spanned 90 days, and digestibility trials were carried out following a month of the feeding trial. The control group exhibited a significantly (*p* < 0.001) lower dry matter intake (DMI) compared to the test feed group, which had a higher intake, particularly in the Ziziphus group. However, the Ziziphus group demonstrated significantly (*p* < 0.01) lower CP digestibility than the other groups. The test diet also led to a significantly (*p* < 0.001) higher weight gain. Notably, the Ziziphus group demonstrated superior performance in weight change (BWC), final body weight (FBW), and average daily gain (ADG). Similar results were observed for CH_4_ production (g/day), CH_4_ yield (g/kg DMI), and CH_4_ intensity (g CH_4_/kg ADG) using both CH_4_ measuring methods. The CH_4_ emission intensity was significantly (*p* < 0.04) lower in the test feed groups than in the control group. The control group emitted 808.7 and 825.3 g of CH_4_, while the Ziziphus group emitted 220 and 265.3 g of CH_4_ per kg of ADG using the Modeling and LMD methods, respectively. This study indicates that LMD could yield biologically plausible data for sheep. Although the small sample size in the Ziziphus group was a limitation of this study, leaf meals from *Ziziphus spina-christi and Acacia nilotica*, which are rich in condensed tannins (CTs), have resulted in considerable weight gain and enhanced feed efficiency, thereby making these leaf meals a viable and sustainable feed option for ruminants in Ethiopia.

## 1 Introduction

In Ethiopian farm households, financial income from sheep and goat production constitutes 40%, which also equates to 19% of the subsistence food value from all livestock production and 25% of the country’s domestic meat consumption (1, 2). Small ruminants also contribute about 2% of the national gross domestic product (GDP) (3). Ethiopia claims an estimated 38 million sheep, 99.6% of which are indigenous breeds. The country also supports 14 traditional sheep-breeding communities (4, 5).

The scenario of sheep production in Ethiopia relies on low input systems such as poor quality and quantity of feed resources, lack of appropriate feeding system, poor production and reproduction traits, and low productive and reproductive performance. This low-productivity production system uses more energy to produce each unit of animal product than those with high-productivity. The low-input system is responsible for the bulk of CH_4_ emissions (6). Based on CSA (4) report, grazing is the primary type of feeding (57.8%), followed by crop residue (29.8%). Hay and by-products comprise about (6.7%) and (1.5%) of the total feeds, with the remaining (4.2%) other feed types, such as improved forage.

Ruminants that consume low-quality feed are known to produce more CH_4_ per unit of product compared to those on higher-quality diets (7). As a result, animal nutritionists are urged to investigate alternative feed resources that can be integrated with existing dietary components to lessen CH_4_ emissions while maintaining productivity (8). Enhanced feeding could significantly improve ruminants’ digestive efficiency and lower CH_4_ emissions by as much as 50% per unit of feed intake (9). In Ethiopia, some of the top feed sources with low CH_4_ yields include *Acacia nilotica (L.)* (6.6 g/kg DM), *Ziziphus spina-christi* (7.8 g/kg DM), and BSG (8.1 g/kg DM) (10).

This study sets out to assess locally available feed that improves the feeding value of the existing feed resource, increases or maintains animal productivity, and reduces CH_4_ intensity (i.e., g CH_4_/kg product) in the local Menz sheep breed in Ethiopia.

The selection criteria for test feeds in this trial were based on the *in vitro* output, focusing on low CH_4_ yield and optimal nutritional content from feed sources available locally in Ethiopia (10). Therefore, the study’s objective was to evaluate the effects of best-bet feeds on enteric CH_4_ emissions, weight gain, digestibility, and methane emission in local Menz sheep breeds. This research supports the country’s goal of adopting climate-smart agriculture within the livestock sector (11).

## 2 Materials and methods

### 2.1 Study area

The experiment was conducted at Debre Berhan Agricultural Research Center (DBARC). The experiment site is located in the central Highlands of Ethiopia about 120 km northeast of Addis Ababa, at an altitude of 2,800 m above sea level. The geographical location of DBARC is from 09°35′ 45″ to 09° 36′ 45″ north latitude and 39° 29′ 40″ to 39°31′ 30″ east longitude.

### 2.2 Experimental animals and management

Twenty-one yearling Menz sheep with a mean initial live body weight of 22.7 ± 1.7 kg (mean ± SD) were purchased from a nearby local livestock market. All sheep purchased were Burdizzo castrated and vaccinated for pasteurellosis, sheep pox, and anthrax during a 15-day quarantine period. In addition, both internal and external parasites were treated with ivermectin.

### 2.3 Experimental feed preparation and feeding

The three test feeds in this experiment include BSG, dried leaves of *Acacia nilotica*, and *Ziziphus spina-Christi.*

*Acacia nilotica* and *Ziziphus spina-christi* leaves were harvested from Debre Berhan University research site and farmers’ trees in Showarobit. The branches were pruned and placed on a canvas to sun dry. Afterward, the leaves were removed by gently striking the branches with sticks. The BSG was obtained from Dashen Brewery factory and sun-dried on a canvas floor. The bulk of the dried foliage and BSG were stored in jute bags for subsequent feeding experiments. The other feed ingredients used for the experiment were Wheat bran (WB), Niger seed cake (NG), and salt lick. Control diet constituted only WB and NG. Supplements were divided into two halves and provided at 8:30 h and 14:00 h. Additionally, grass hay, dominated by *Andropogon amethystinus Steud hay*, was chopped manually and fed *ad libtum* along with salt lick as a basal diet to all animals. Water was available freely, daily feed offered, and refusals were recorded.

### 2.4 Experimental treatments and design

The treatment feeds were calibrated such that the test feeds contained equivalent amounts of crude protein to the control diet. The feeding experiment was conducted using a randomized complete block design for 90 days. Six animals were assigned to each treatment, except for the Ziziphus group, which had only three animals due to a shortage of *Ziziphus spina-christi* at the time of the experiment. A possible limitation of the study is that only three sheep were used for the Ziziphus group, and one animal from the BSG group had to be dropped due to a sudden unexplained drop in intake.

### 2.5 Data collection

#### 2.5.1 Feed intake measurement

The diet offered, and orts were recorded daily for each animal to measure DMI. Representative samples of feed offered per batch and refused per animal were collected every 3 days to determine dry matter.

#### 2.5.2 Live weight measurement

Initial body weight was measured by taking the mean of two consecutive weights after overnight fasting before the beginning of the actual feeding trial and every 15 days thereafter using hanging digital balance with 10 g graduation.

#### 2.5.3 Apparent digestibility trial

At 31 days of the feeding trial, animals were fitted with fecal collection bags for digestibility study for 10 consecutive days, where the first 3 days were used as adaptation and the remaining 7 days as sampling. All the daily fecal outputs were collected, weighed, and recorded for each animal, and a sub-sample of approximately 100 g was taken from each animal after thorough mixing and stored at - 20°C. The frozen daily fecal output was thawed, pooled for the sampling days per animal and treatment, and dried at 65°C for 72 h. Dried samples were ground to pass through a 1 mm sieve and stored in a plastic bag for chemical analysis.

### 2.6 Enteric CH_4_ measurements

**Figure 1:**
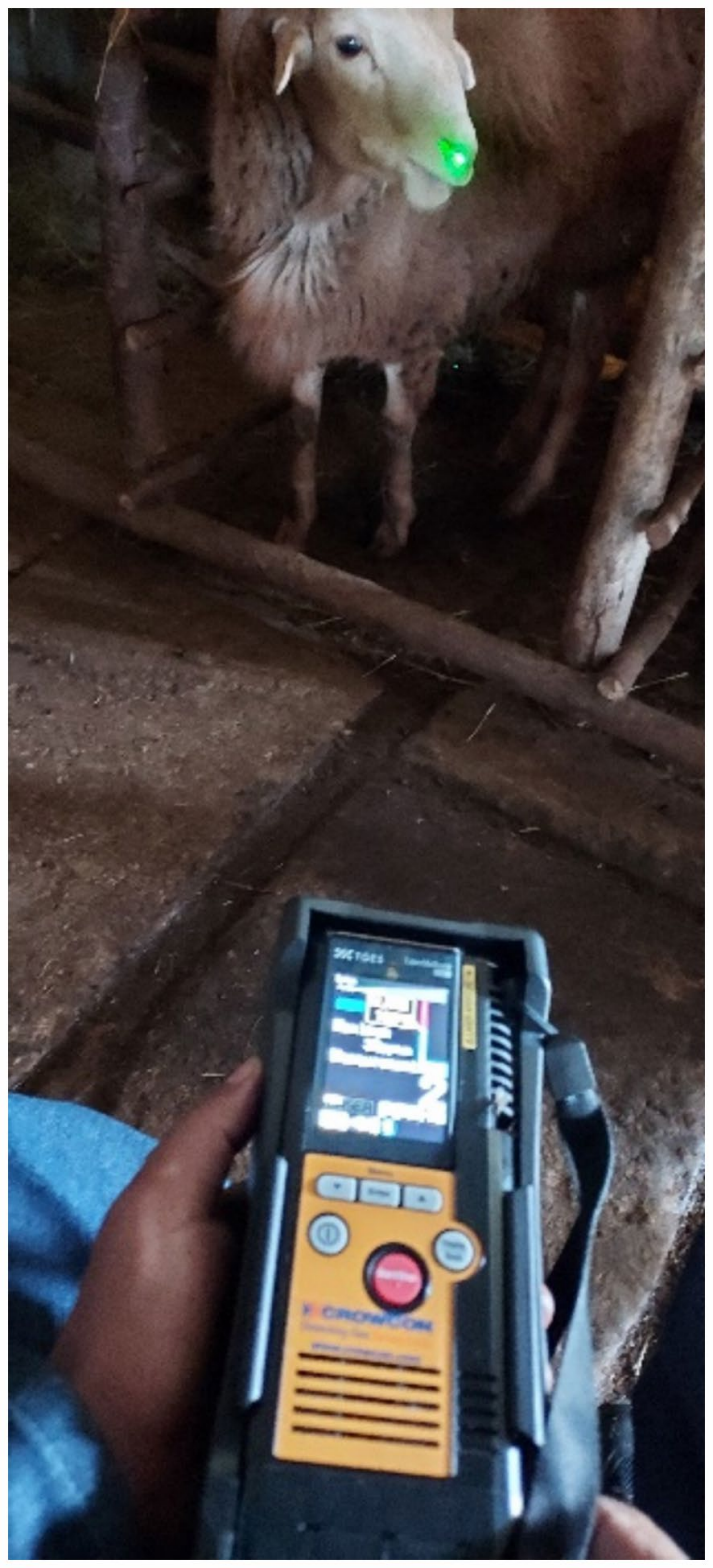
Demonstrating LMD measurement during the experiment.

In the final week of the experiment, a portable laser CH_4_ detector LMD (Crowcon Detection Instruments Ltd., Tokyo, Japan), was utilized for three consecutive days before and after the morning and evening feedings. The concentration of CH_4_ was measured by directing a green laser beam toward the nostrils of the sheep for 3 continuous minutes to estimate the CH_4_ concentration at 1 m from the resting animal in their feeding pen (Figure 1). The LMD was linked to a tablet with the GasViewer app through Bluetooth for data export and storage. The LMD’s output is a time series of CH_4_ emission values from a single animal, encompassing both eructation and respiration, indicative of the respiratory cycle. The nonlinear generalized reduced gradient (nonlinear GRG) method in Excel 2019 (Microsoft Corporation, Redmond, WA, USA) was employed for calculating eructation and respiration, as documented by Kobayashi et al. (12). The CH_4_ levels, recorded in parts per million (ppm), were then converted to grams per day using a formula adapted from Lanzoni et al. (13), specifically tailored for sheep.

$$CH4\left(\frac{g}{\min}\right)=CH4\;\mathrm{average}*V*R*\alpha *\beta *10^{-6}$$

$$CH4\left(\frac{g}{\mathrm{day}}\right)=CH4\left(\frac{g}{\min}\right)*1440\left(\frac{\min}{\mathrm{day}}\right)$$

Where, V represents the tidal volume (which is 12 mL/kg body weight); R denotes the respiratory rate (resting respiratory rate of sheep ranges from 16 to 34 breaths per minute according to Reece et al. (14), and an average of 25/min is used); *α* is the conversion factor for (CH_4_ 0.000667 g/mL); and, *β* denotes factor representing the dilution correction, (which accounts for the discrepancy between breath and total methane production in sheep and typically assumed to range from 5 to 8, with an average value of 6.5 being adopted). The value 1,440 represents the number of minutes per day. The CH_4_ average utilized in this study represents the mean of peaks and troughs, aiding in capturing each respiratory cycle (15). In addition, the current dilution factor assumed the difference in tidal volume, CH_4_ production, and respiratory rate between sheep and large ruminants.

In addition, enteric CH_4_ emissions were estimated using a model developed from an intercontinental database (16). Using the following formula:


$$CH4\;\left(\frac{g}{\mathrm{day}}\right)=-0.669+9.19*DMI+0.0495*OMD+0.169*BW$$

Where DMI denotes dry matter intake, OMD denotes organic matter digestibility, and BW denotes body weight.

### 2.7 Chemical analysis

The chemical composition of the feed ingredients is presented in (Table 1) and the proportion of the treatment feed ingredients (Table 2). A detailed procedure, including *in vitro* CH_4_ yield and condensed tannin content (CTs), of the test feed can be found in Bekele et al. (10).

**Table 1:** Chemical composition of the experimental feed ingredient used for *in vivo* feeding study.

Feed ingrident	DM, g/kg	Chemical composition (g/kg DM)			Total CTs in mg/g
		NDF	CP	OM	
*Grass hay (Andropogon amethystinus Steud)*	944	652	46	916	–
*Acacia nilotica (L.)*	946	142	204	940	15.3
*Ziziphus spina-christi*	927	254	158	918	340.8
Brewery’s spent grain	934	519	253	957	–
Wheat bran	923	380	166	942	–
Niger seed cake	940	191	442	924	–

NDF: NDF assayed with a heat-stable amylase and expressed inclusive of residual ash; CP: crude protein; DM: dry matter; OM: organic matter; CTs: condensed tannins including soluble and cell-bound. Source: Bekele et al. (10).

**Table 2:** Proportion of the treatment feed ingredients on a DM basis for the *in vivo* trial.

Treatments	Wheat bran (g)	Noug seed cake (g)	Test feed (g)	Grass hay(g)
Control	200	200	0	*Ad libitum*
Acacia	100	100	304	*Ad libitum*
BSG	100	100	243.2	*Ad libitum*
Ziziphus	100	100	380	*Ad libitum*

### 2.8 Calculations for derived values

Daily feed intake (g/d) of both supplement and basal feeds was determined by the difference between the amount of feed offered and its constituents (OM, CP, NDF) and refusal on a dry matter basis.

The average daily live weight gain (ADG, g/d) was calculated by subtracting initial weight from final weight and dividing by the days of feeding.

Apparent total tract digestibility coefficients of feed DM and its nutrients were calculated from the ingested and excreted amounts in the feces of each component.

Additionally, calculations were performed to derive data for protein efficiency ratio (PER) and CH_4_ emission intensity from the literature that did not provide direct values.

### 2.9 Data analysis

The analysis of variance (ANOVA) for data on feed intake, body weight change (BWC), and digestibility was conducted using R version 4.3.0 (17). Initial body weight (IBW) served as a covariate in the statistical analysis of ADG, BWC, and final body weight (FBW). This was performed using the ‘lm’ function with the following model:


$$Y_{ij}=\mu +T_{i}+B_{j}+e_{ij}$$

Where

Y_ij_ is an observed variable for the i^th^ treatment, j^th^ block. μ is the overall mean, T_i_ is i^th^ treatment, B_j_ is j^th^ block, and e_ij_ is the residual error for the i^th^ treatment and j^th^ block. The results were considered statistically significant when *p* < 0.05. Duncan’s Multiple Range Test method was employed for *post hoc* analysis. Simple linear regression analysis was also performed using the “ggscatter” function from the “ggpubr” package.

## 3 Results

### 3.1 Feed intake and apparent digestibility

The Ziziphus group demonstrated significantly (*p* < 0.001) higher DM and OM intake than others, while the BSG group showed the highest NDF intake. Additionally, the Ziziphus group exhibited the lowest CP digestibility (Table 3).

**Table 3:** Dry matter, nutrient intake (g) and digestibility coefficient of Menz sheep fed natural pasture hay as a basal diet and supplemented with control and test feed.

Intake (g)	Treatments				SEM	*p*-value
	Control	Acacia	BSG	Ziziphus		
DM	1085^d^	1185^b^	1124^c^	1254^a^	3.1	0.001
NDF	569^c^	558^d^	633^a^	607^b^	1.7	0.001
CP	154	157	155	155	0.46	0.14
OM	997^c^	1093^b^	762^d^	1150^a^	2.4	0.001
Digestibility						
DM	0.68	0.70	0.66	0.64	0.01	0.5
NDF	0.60	0.63	0.62	0.57	0.02	0.75
CP	0.81^a^	0.77^a^	0.79^a^	0.67^b^	0.01	0.003
OM	0.70	0.72	0.68	0.67	0.01	0.5

NDF, NDF assayed with a heat-stable amylase and expressed inclusive of residual ash; CP, crude protein; OM, organic matter: ^a,b,c,d^Mean values within a row with different superscript letters differ significantly (*p* < 0.05). Supplement: Niger seed cake, wheat bran, brewery-spent grain, and test feed.

### 3.2 Body weight change

**Figure 2:**
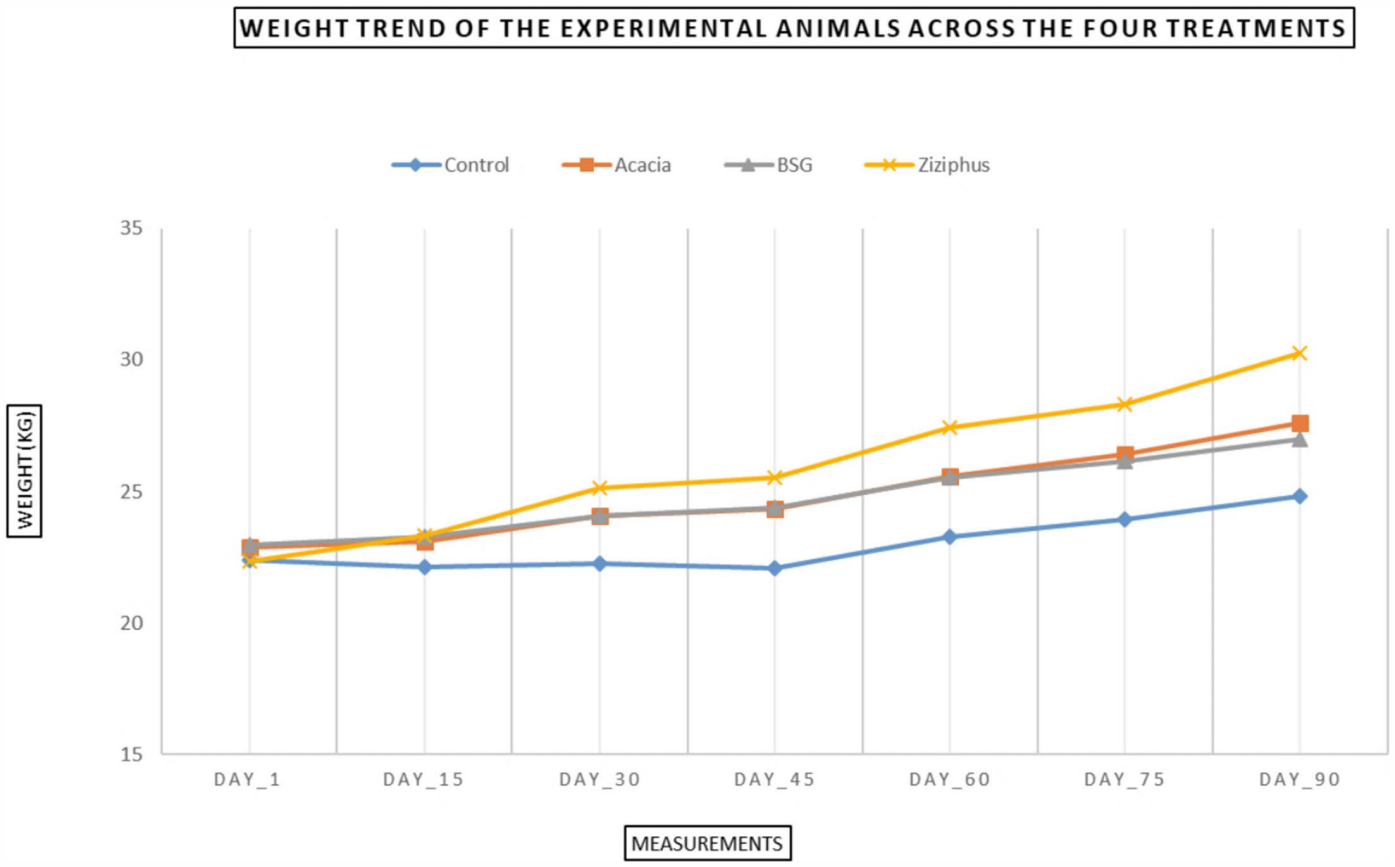
Trend in weight change over 90 days of the experimental period for Menz sheep.

The test diet groups resulted in significantly more ADG than the control group (Table 4). Among the treatments, the Ziziphus group showed significantly (*p* < 0.001) the highest ADG, FE, and PER. Additionally, sheep fed with dried leaves (Ziziphus and Acacia groups) had significantly higher ADG than those solely on agro-industrial by-products (BSG and Control groups), as shown by the weight trend in Figure 2.

**Table 4:** Body weight change, feed efficiency and protein efficiency ratio of Menz sheep fed natural pasture hay as basal diet and supplemented with control and test feed.

Variables	Treatments				SEM	*p*-value
	Control	Acacia	BSG	Ziziphus		
IBW(kg)	22.4	22.9	22.8	22.4	0.20	0.7
FBW(kg)	24.8^c^	27.6^b^	27.0^b^	30.3^a^	0.33	0.001
BWC(kg)	2.4^c^	4.7^b^	4.1b^c^	7.9^a^	0.29	0.001
ADG(g)	27.0^c^	52.4^b^	45.1^bc^	87.7^a^	3.15	0.001
FE (%)	2.5^c^	4.5^b^	4.0^bc^	7.0^a^	0.28	0.003
FCR	52^a^	23.4^b^	27.4^ab^	14.3^b^	4.14	0.04
PER	0.17^c^	0.33^b^	0.29^bc^	0.57^a^	0.020	0.001

IBW, initial body weight; FBW, final body weight; BWC, body weight change after feeding supplement (FBW-IBW); ADG, average daily gain; FE, feed efficiency calculated as average daily gain (ADG)/DMI; PER, protein efficiency ratio calculated as ADG/CP intake: ^a,b,c^Mean values within a row with different superscript letters differ significantly (*p* < 0.05).

### 3.3 Enteric CH_4_ emission

**Figure 3:**
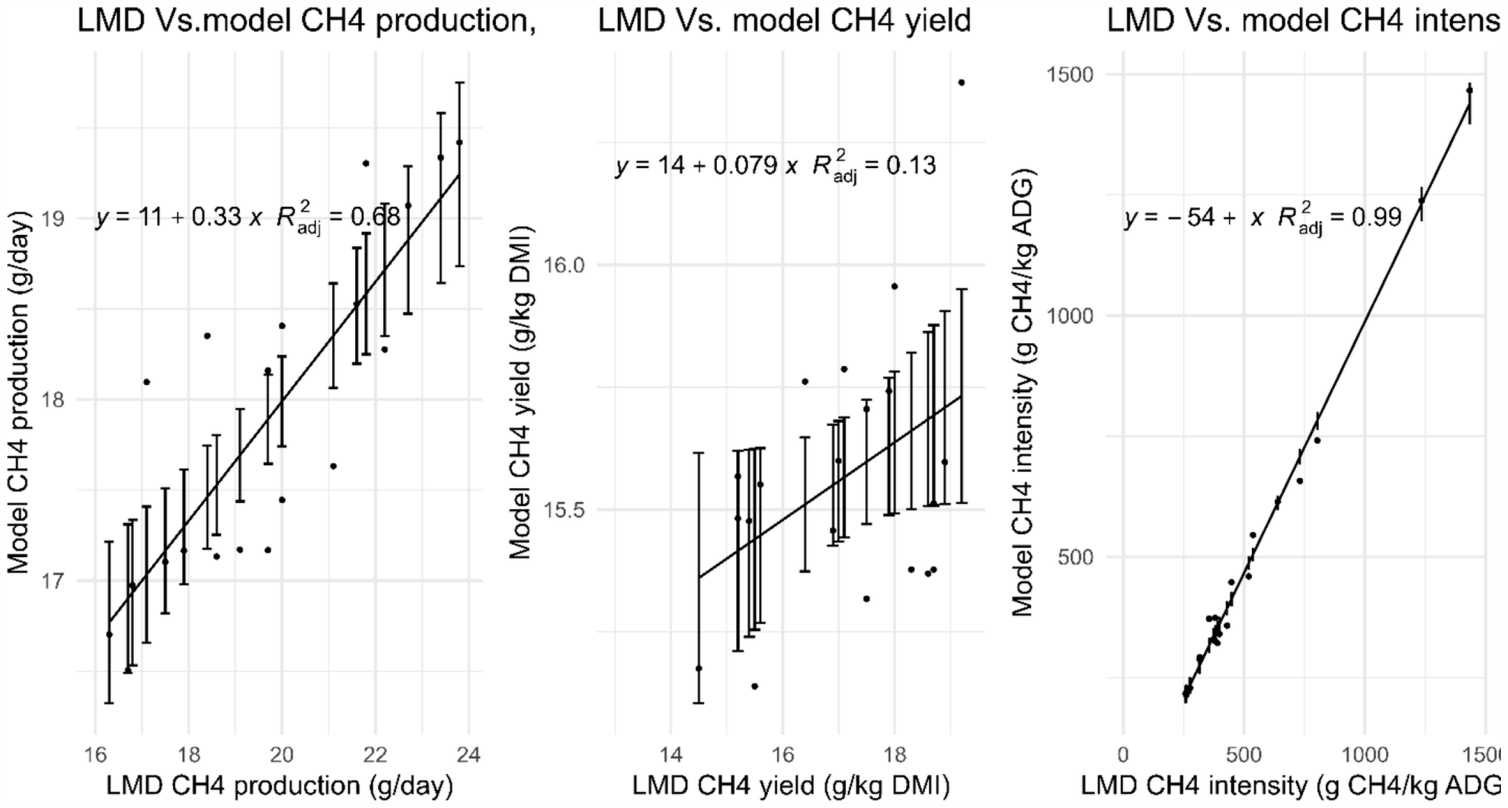
Relationship between enteric CH_4_ emissions measured by LMD Vs. Modeling from local Menz sheep breed in Ethiopia.

**Figure 4:**
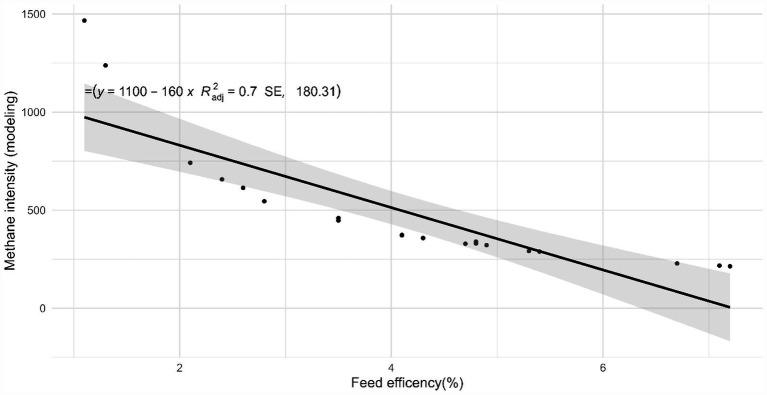
Feed efficiency effect on CH_4_ intensity by the Menz sheep.

**Figure 5:**
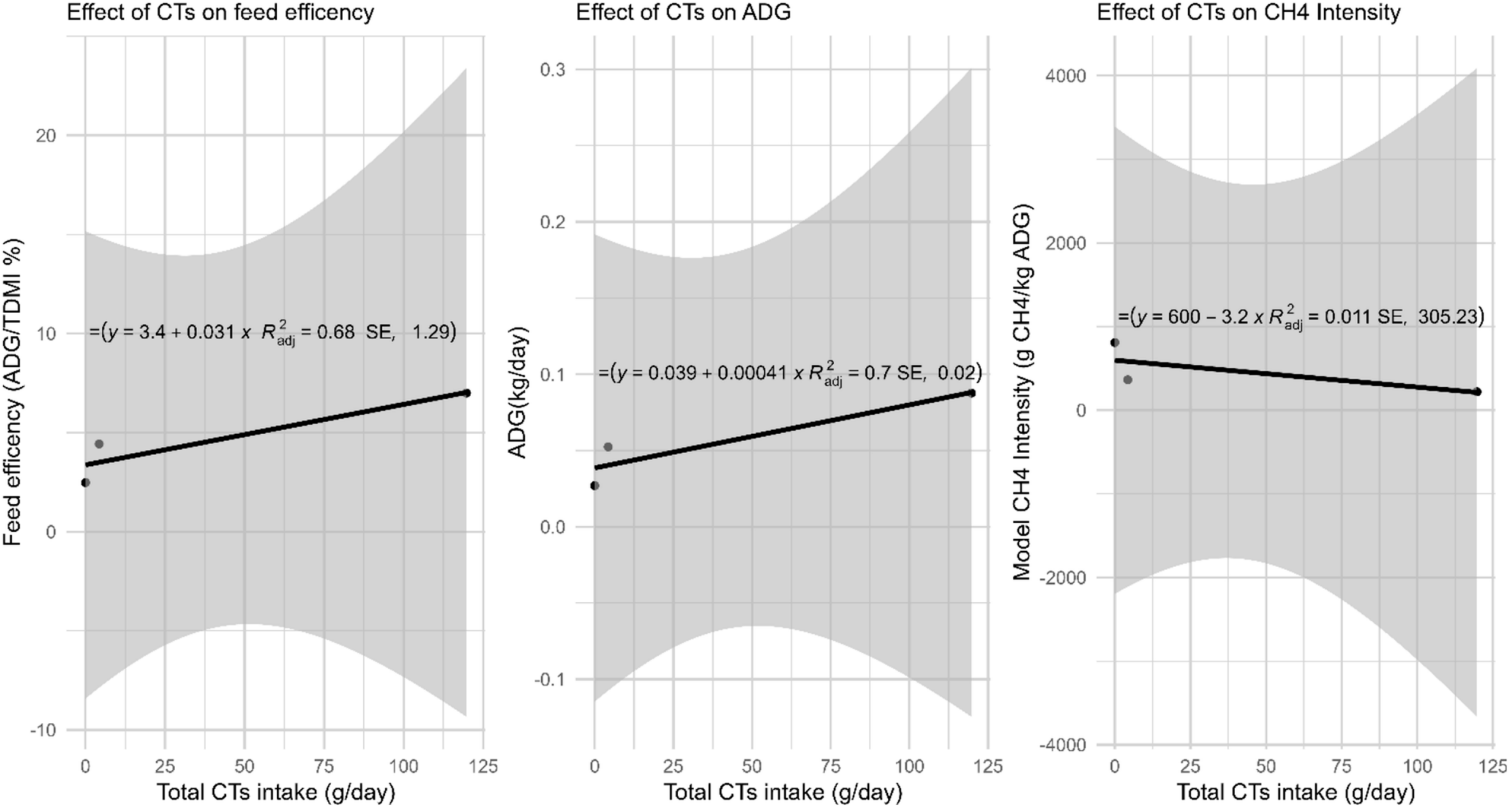
The effect of CTs intake on feed efficiency, average daily gain and CH_4_ intensity of Menz Sheep (Average of FE, ADG and CH_4_ intensity from modelling and total CTs values were used from Control, Acacia and Ziziphus group to construct the scatter plot).

Table 5 presents the estimated enteric CH_4_ emissions derived from modeling and LMD methods. The Ziziphus group recorded the highest CH_4_ production yet the lowest CH_4_ intensity among all treatment groups. Conversely, the control group had the lowest CH_4_ production but the highest CH_4_ intensity according to both methods. The results from the LMD and modeling were consistent across treatments, showing a strong correlation between CH_4_ production and intensity, as evidenced by high (*R*^2^_adj_ = 0.68–0.99), as depicted in Figure 3. This study found that feed efficiency (FE) significantly influenced CH_4_ intensity, and the effect of FE on CH_4_ intensity was greater than that of CT intake. The optimal *R*^2^_adj_ value for FE was 0.70 (Figure 4), whereas the R^2^_adj_ for total CT intake was only 0.011 (Figure 5).

**Table 5:** Enteric CH_4_ production, yield, and intensity of Menz sheep fed natural pasture hay as basal diet and supplemented with control and test feed using modeling and laser CH_4_ detector methods.

Enteric CH_4_	Treatments				SEM	*p*-value
	Control	Acacia	BSG	Ziziphus		
CH_4_ production (g/day)						
Modeling	17^d^	18.4^b^	17.6^c^	19.3^a^	0.06	0.001
LMD	17.8^c^	20.0^b^	19.8^b^	23.3^a^	0.29	0.001
Average	17.4	19.2	18.7	21.3		
SD	0.49	1.06	1.56	2.83		
CV (%)	3	6	8	13		
CH4 yield (g/kg DMI)						
Modeling	15.6	15.6	15.6	15.4	0.04	0.25
LMD	16.3^b^	16.8^b^	17.6^ab^	18.5^a^	0.23	0.04
Average	16.0	16.2	16.6	17		
SD	0.49	0.85	1.4	2.2		
CV (%)	3	5	9	13		
CH4 intensity (g CH4/kg ADG)						
Modeling	808.7^a^	363.5^b^	428.4^ab^	220^b^	64.41	0.04
LMD	825.3^a^	391^b^	478^ab^	265.3^b^	61.51	0.04
Average	817	377.3	453.1	242.7		
SD	11.7	19.4	34.9	32		
CV (%)	1	5	8	13		

CH_4_, methane; DMI, dry matter intake; ADG, average daily gain; ^a,b,c,d^Mean values within a row with different superscript letters differ significantly (*p* < 0.05).

Figure 5 illustrates the impact of CTs on FE, ADG, and CH_4_ intensity. An incremental increase in total CT intake correlates with a 0.031% increase in FE and a 0.41 g/d rise in ADG. Moreover, CH_4_ intensity is reduced by 3.2 grams per day.

## 4 Discussion

In this *in vivo* study, we employed modeling and LMD methods to estimate enteric CH_4_ emissions. Furthermore, the trial results indicated that CT intake affects ADG, FE, and CH_4_ emissions. In addition, FE had a notable effect on weight gain and CH_4_ intensity.

### 4.1 Feed intake and apparent digestibility

The experimental diet, designed to be iso-nitrogenous, led to a notably higher consumption of *Ziziphus spina-christi dried leaves* in the Ziziphus group. Consequently, this group showed a significantly (*p* < 0.001) greater supplement and total DMI. This is attributed to the bulkiness of *Ziziphus spina-christi* compared to the other treatment groups.

The research indicated that there were no significant differences (*p* > 0.05) in the intake of crude protein (CP) among the treatment groups, and the average daily consumption of CP met the satisfactory levels as defined by Kearl (18) and NRC (19). Nevertheless, OM, FE, and PER were significantly (*p* < 0.001) higher in the Ziziphus group compared to other treatments. The protein efficiency ratio in this study ranged from 0.18 to 0.57, which is lower than the ratios found in afar sheep fed varying levels of *Brassica carinata* cake (0.65–0.83) (20) and Menze sheep supplemented with tagasaste leaves (0.73 to 0.91). However, the Ziziphus group showed a comparable PER value to goats supplemented with *Ziziphus spina-christi* leaves in Ethiopia (21).

The higher PER in the Ziziphus group may be attributed to the higher levels of bypass protein available. Valizadeh et al. (22) observed that lambs fed with low, medium, and high levels of bypass protein exhibited increased ADG and PER proportional to the bypass protein levels. Additionally, the notable PER in the Ziziphus group could stem from the increased DMI, and the beneficial effects of tannins on nitrogen utilization efficiency. Orzuna-Orzuna et al. (23) reported that in ruminants, dietary supplementation of CTs enhances the efficiency of ingested feed by increasing the duodenal flow of microbial protein and amino acids without losses in the rumen (24, 25). Protein Efficiency Ratio measures the nutritive value of protein sources. The higher the PER value of a protein, the more beneficial it is to the animal. It is also the easiest method of assessing the quality of proteins (26).

The group fed with Ziziphus exhibited significantly (*p* < 0.01) lower CP digestibility when compared to other groups. This difference is likely attributed to the high levels of CT present in the Ziziphus group. Kumar (62) noted that condensed tannins significantly affect digestibility, yet the influence of CT on rumen digestion differs depending on their concentration, type, and activity. Additionally, except for BSG, agro-industrial by-products typically have low fiber content and high digestibility, as noted by Mengistu et al. (27).

### 4.2 Body weight change

Supplements sourced from indigenous plants significantly (*p* < 0.001) enhanced BWC and ADG compared to the Control and BSG groups. This effect may be attributed to the beneficial role of secondary plant metabolites such as CTs (28). Min et al. (29) noted that moderate levels of tannins in forage legumes offer multiple benefits for ruminants, such as improved growth rates. Reducing rumen forage protein degradation due to reversible binding to these proteins and reducing the populations of proteolytic rumen bacteria increases essential amino acid absorption from the small intestine.

In the current *in vivo* study, the ADG ranged from 27 g/day for the control group to 87 g/day for the Ziziphus group. This resulted in a corresponding WC of 2.4 kg to 7.9 kg, respectively, over a period of 90 days during the feeding trial. The Ziziphus group showed superior results compared to similar research conducted elsewhere in Ethiopia. For example, Hailecherkos et al. (30) found that supplementing tree lucerne (*Chamaecytisus palmensis*) dried leaves or concentrate mixture to Washera sheep resulted in an ADG of 51.1 to 82.2 g/day. Worku et al. (31) reported that supplementing Kafa sheep with rice bran, Sesbania (*Sesbania sesban*) leaf, and their mixtures resulted in an ADG of 42.4 to 86.1 g/day. Kokeb et al. (32) found that supplementing Dorper-Menz crossbred sheep fed with local brewery by-product (*Atella*) and concentrate mixture led to an ADG of 42.2 to 73.2 g/day. Bonsi et al. (33) studied the effect of protein supplement sources (cottonseed cake, sundried leaves of *Leucaena leucocephala*, and sundried leaves of *Sesbania sesban*) on Menz sheep and found an ADG of 32.6 to 62.9 g/day. Our findings showed lower results compared to Ali et al. (34), who reported an ADG of 46.7 to 190 g/day for Bati goat breeds in Ethiopia when supplemented with sun-dried *Ziziphus spina-christi*. Mangara (35) also observed superior weight gain in goats fed with *Ziziphus spina-christi* than those fed with *Combretum adenogonium.* The enhanced performance of the Ziziphus group may be attributed to its high CT content (10). Basyony et al. (36) suggested that *Ziziphus spina-christi* leaves could be used as a natural growth promoter in rabbit diets. Its active ingredients have been shown to have antibacterial and antifungal properties (37).

### 4.3 Enteric CH_4_ emission from sheep

Several methods have been developed to measure CH₄ emissions from ruminants. All methods have different scopes of applications, advantages, and disadvantages, and none of them is perfect in all aspects. Respiration chambers yield the most accurate measures of total enteric CH_4_ emissions from ruminants. Modeling and LMD are non-invasive, non-contact, user-friendly, and cost-effective methods compared to the respiration chamber, rendering them suitable for resource-constrained countries such as Ethiopia (38–40).

#### 4.3.1 Methane production

Comparable results were observed from the LMD values and reports of other studies using respiration chambers. For instance, Pelchen and Peters (41) found 22.2 g/day from 1,137 sheep observations under various feeding conditions. Pinares-Patiño et al. (40) reported 22.7 g/day in sheep-fed grass and 18.6 g/day in those fed pellets. The CH_4_ production from modeling in our study was lower than that reported by Belanche et al. (16), which was 19.9 g/kg of sheep from an intercontinental database. However, it was higher than Amaral et al. (42), who reported 10.9–15.5 g/day for sheep grazing on pearl millet swards, measured using the sulfur hexafluoride (SF6) tracer technique. Chagunda et al. (43) reported that ruminating cows produced higher CH_4_ production using LMD than estimated by empirical modeling, which aligns with our findings. In a separate study, Chagunda et al. (44) found that LMD measurements showed higher means and variation than those taken from metabolic chambers in sheep and cattle. However, the trend of the measurements from the LMD and the metabolic chamber was similar. Therefore, LMD could be a plausible technique for CH_4_ emission studies in Sheep if a large number of animal data is taken.

This study observed that higher total intake relates to increased CH_4_ production in both CH_4_ estimation methods. According to Gebbels et al. (45), the main factor leading to increased net CH_4_ emissions in sheep meat and wool enterprises is the rise in total feed intake. In line with this, Patra et al. (46) identified feed intake as the most significant predictor of CH_4_ production in sheep. Belanche et al. (16) also highlighted the importance of DMI for predicting enteric CH_4_ emissions in sheep where DMI alone explains 80–91% of the variation in CH_4_ production in sheep (47). Consistent with our findings on identifying treatment differences using LMD methods, Kang et al. (48) also detected variations in CH_4_ emissions from cattle based on forage intake levels utilizing the LMD method. They recommended its application for assessing the effects of dietary treatments on CH_4_ concentrations in cattle.

#### 4.3.2 Methane yield

CH_4_ yield reflects the methanogenic potential of the digestive process and correlates with CH_4_ production (49). In the current study, the LMD CH_4_ yield ranged from 16.3 to 18.5 g CH_4_/kg DMI, the highest observed in the Ziziphus group. The higher CH_4_ yield in the Ziziphus group may stem from variations in the feed’s chemical composition and degradability (50, 51). Starsmore et al. (52) stated that DMI influences CH_4_ emissions. Higher DMI provides more material for fermentation in the rumen, which is positively linked to CH_4_ emissions.

Washaya et al. (53) fed Xhosa lop-eared goats in South Africa a forage having secondary metabolites such as tannins, phenolic, and saponins and measured the CH_4_ using LMD and found CH_4_ yield of 12.6 to 13.1 g/kg DMI. Waghorn et al. (54) conducted indoor trials with sheep in metabolism crates and observed CH_4_ yields ranging from 11.5 g CH_4_/kg DMI with lotus to 25.7 g CH_4_/kg DMI with pasture. The current *in vivo* study contradicted our previous *in vitro* findings, showing a low CH_4_ yield from the indigenous plant feed source (10). Any *in vitro* methodology and batch culture are handy when evaluating treatments, feeds, or additives. They provide a quick response to elucidate a treatment’s potential impact on fermentation. However, they cannot necessarily be directly applied to make assumptions of responses *in vivo* (55). The most accurate way to evaluate the nutritional value of any feedstuff is to feed it to the appropriate class of animal using feeding trials, which is the standard measure of digestibility (56).

#### 4.3.3 Methane emission intensity

Methane intensity strongly depends on milk or meat production output (49). Savian et al. (57) and Silva et al. (58) observed that CH_4_ emissions intensity from grazing sheep varied from 159 to 285 g CH_4_/kg ADG, and from young bulls consuming soybean lipids, it ranged between 105 to 169 g CH_4_/kg ADG, respectively, as measured by the SF6 tracer technique. El-Zaiat et al. (59) also observed a range of 93 to 131 g CH_4_/kg ADG in lambs supplemented with encapsulated nitrate and cashew nutshell liquid, using open-circuit respiration chambers for measurement.

The higher CH_4_ emission intensity observed in our study could be attributed to the lower fattening efficiency of the Menze breed. Indigenous sheep breeds in Ethiopia, particularly the Menz breed, are often regarded as low-producers, with the Menz breed being notably slow-growing (60). Kurihara et al. (61) highlighted the variation in CH_4_ emission intensity associated with live weight gain, diet quality, and fattening efficiency.

## 5 Conclusion

In this research, the test feed outperformed the control group in terms of body weight gain and CH_4_ emission intensity in sheep. The Ziziphus group notably showed a significantly greater increase in final body weight. The average enteric CH_4_ emission, as measured by the two methods, displayed concordance in all CH_4_ variables, such as CH_4_ production and CH_4_ intensity, as evidenced by strong R^2^. A laser CH_4_ detector could potentially estimate CH_4_ in sheep where there is no access to other measurement equipment. It is also a friendly and economical method for estimating CH_4_ in a country like Ethiopia. Methane emission intensity is the ideal variable related to the fattening efficiency of the sheep. Furthermore, possibly due to secondary metabolites such as CTs, the Acacia and Ziziphus groups exhibited optimal body weight gain in sheep compared to the control diet, suggesting their suitability for sustainable ruminant production in tropical regions. This study identified that the indigenous Menz breed sheep have low fattening efficiency, highlighting the need for breed improvement. In our results, feed efficiency promotes better weight gain, which leads to lower CH_4_ per unit of average daily gain. In conclusion, supporting research and extension services to promote the utilization of leaf meals in the diet of ruminant livestock is a sustainable feeding option in Ethiopia.

## Data Availability

The raw data supporting the conclusions of this article will be made available by the authors, without undue reservation.
